# Replication of obesity and diabetes-related SNP associations in individuals from Yucatán, México

**DOI:** 10.3389/fgene.2014.00380

**Published:** 2014-11-18

**Authors:** Victor M. Hernandez-Escalante, Edna J. Nava-Gonzalez, V. Saroja Voruganti, Jack W. Kent, Karin Haack, Hugo A. Laviada-Molina, Fernanda Molina-Segui, Esther C. Gallegos-Cabriales, Juan Carlos Lopez-Alvarenga, Shelley A. Cole, Marguerite J. Mezzles, Anthony G. Comuzzie, Raul A. Bastarrachea

**Affiliations:** ^1^Facultad de Medicina, Merida, Universidad Autonoma de YucatanYucatan, Mexico; ^2^Facultad de Salud Publica y Nutricion, Universidad Autonoma de Nuevo LeonNuevo Leon, Monterrey, Mexico; ^3^Nutrition and UNC Nutrition Research Institute, University of North CarolinaChapel Hill, NC, USA; ^4^Department of Genetics, Texas Biomedical Research InstituteSan Antonio, TX, USA; ^5^Departamento de Investigación, Escuela de Ciencias de la Salud, Universidad Marista de MeridaMerida, Yucatan, Mexico; ^6^Facultdad de Enfermeria, Universidad Autonoma de Nuevo LeonNuevo Leon, Monterrey, Mexico

**Keywords:** Single Nucleotide Polymorphisms (SNP), association analysis, minor allele frequency, type 2 diabetes, obesity

## Abstract

The prevalence of type 2 diabetes (T2D) is rising rapidly and in Mexicans is ~19%. T2D is affected by both environmental and genetic factors. Although specific genes have been implicated in T2D risk few of these findings are confirmed in studies of Mexican subjects. Our aim was to replicate associations of 39 single nucleotide polymorphisms (SNPs) from 10 genes with T2D-related phenotypes in a community-based Mexican cohort. Unrelated individuals (*n* = 259) living in southeastern Mexico were enrolled in the study based at the University of Yucatan School of Medicine in Merida. Phenotypes measured included anthropometric measurements, circulating levels of adipose tissue endocrine factors (leptin, adiponectin, pro-inflammatory cytokines), and insulin, glucose, and blood pressure. Association analyses were conducted by measured genotype analysis implemented in SOLAR, adapted for unrelated individuals. SNP Minor allele frequencies ranged from 2.2 to 48.6%. Nominal associations were found for CNR1, SLC30A8, GCK, and PCSK1 SNPs with systolic blood pressure, insulin and glucose, and for CNR1, SLC30A8, KCNJ11, and PCSK1 SNPs with adiponectin and leptin (*p* < 0.05). *P*-values greater than 0.0014 were considered significant. Association of SNPs rs10485170 of CNR1 and rs5215 of KCNJ11 with adiponectin and leptin, respectively, reached near significance (*p* = 0.002). Significant association (*p* = 0.001) was observed between plasma leptin and rs5219 of KCNJ11.

## Introduction

Epidemiological studies have shown that obesity is associated with an increased risk of mortality (Flegal et al., 2007) including via increased risk of T2D and cardiovascular disease (Ogden et al., 2007). Obesity and T2D have strong genetic components (Rankinen et al., 2006; Sandholt et al., 2010). Recent genome-wide association studies (GWAS) have identified single nucleotide polymorphisms (SNPs) in or near a large number of genes that are related to obesity and T2D phenotypes (Tung and Yeo, 2011). The mechanism by which variation in these genes influences body weight and biomarkers of glucose metabolism is not yet known.

The prevalence of T2D in Mexico in 2000 was 10.9%. By the year 2012 this prevalence almost doubled to 19%. The prevalence of being overweight or obese (BMI ≥ 25 kg/m^2^) is higher in Mexican women (73.0%) than men (69.4%) similar to obesity alone (BMI ≥ 30 kg/m^2^) (Gutierrez et al., 2012). These national statistics are reflected in the regions within the country, including the state of Yucatán. Strong predictors of diabetes included a family history of the disease, an increased body mass index, elevated liver-enzyme levels, smoking status, and reduced measures of insulin secretion and action.

As our study subjects are from the Yucatán peninsula, we are interested in the results from a significant study on the state of health in the population of Mérida, Yucatán performed in 1999, which revealed some noteworthy statistics when compared to the prevalence for metabolic and cardiovascular risk factors around Mexico documented in 2000. For adults between 20 and 75 years of age, 45% of men and 73% of women were overweight (BMI ≥ 27.8 for men and ≥ 27.3 for women), and 19.5% of men and 41.1% of women were obese (BMI ≥ 30). T2D was seen in 6.1% of men and 7.4% of women (Arroyo et al., 1999). This may reflect a particular trend for a relatively higher index of these risk factors in the general population of the Yucatán compared to the rest of México as it is seen in more recent epidemiologic studies (Arroyo et al., 2007; Gutierrez et al., 2012). Therefore, intrinsic genetic factors should not be ignored as the Yucatán population has a very unique Maya-Hispanic racial admixture that has experienced very little out-migration.

The genetic factors involved in the development of obesity and T2D include SNPs in genes that encode proteins influencing body composition as well as fat and glucose metabolism (Hirschhorn and Daly, 2005). These includes those influencing: (a) fatty acid oxidation and glucose uptake by adiponectin through increased AMPK and PPAR-α ligand activities (*ADIPOR1*, *ADIPOR2*) (Yamauchi et al., 2003; Crimmins and Martin, 2007), (b) proinsulin and proglucagon processing and cleavage (*PCSK1*) (Choquet et al., 2013), (c) activation of the CB_1_ receptor to increase de novo lipogenesis in the liver (*CNR1*) (Osei-Hyiaman et al., 2005), (d) stimulation of the Wnt signaling pathway with activation of B-catenin and target genes in the nucleus, repressing proglucagon synthesis in enteroendocrine cells (*TCF7L2*) (Jin and Liu, 2008), (e) regulation of glucose metabolism in liver and pancreas (*GCK*) (Wang et al., 2013), (f) a K_ATP_ channel that plays a major role in insulin secretion (*KCNJ11*) (Olson and Terzic, 2010), and (g) regulation of insulin secretion in humans through a pancreatic β-cell-specific zinc transporter (*SLC30A8*) (Chimienti et al., 2006). These genes were carefully selected due to their possible role in the etiology of obesity and T2D, in addition to their association with insulin-mediated glucose profile and an abnormal expression of adipose tissue endocrine and inflammatory factors. Such parameters have been well-documented as obesity-related metabolic complication leading to cardiovascular pathology and endothelial dysfunction.

Chronic-degenerative and metabolic diseases related to nutrition, lifestyle changes, and genetic predisposition are increasing at alarming rates. Diseases such as arterial hypertension, T2D, dislipidemias, and obesity, all traditionally viewed as risk factors for coronary heart disease and cerebrovascular disease, now constitute a rapidly advancing, world-wide epidemic (Roger et al., 2011). Many studies indicate that México is in the midst of an epidemiological transition that began in the 1950s. It is experiencing a dramatic decrease in mortality from infectious diseases and a sustained increase in mortality from chronic-degenerative diseases (Rivera et al., 2002). National statistics show that the primary cause of general mortality in Mexico is heart disease (Sanchez-Castillo et al., 2005). These nationally representative surveys are reflected in the regions within the country. Since 1990, the primary cause of death in the adult population in Yucatan has been heart disease (Arroyo et al., 2007). Studies of cardiovascular risk factor's prevalence conducted in the Yucatan, also indicate that the prevalence of such conditions are significantly present in this population when compared to those reported in such Mexican national surveys (Arroyo et al., 1999).

In the present study we hypothesize that a population of unrelated individuals from the Yucatan, sharing a common cultural environment and perhaps peculiar intrinsic genetic factors, may help detect associations between obesity and type 2-related genes and risk factor phenotypes. Thus, a community-based cohort from the Yucatan Peninsula was utilized to examine the associations between the genetic polymorphisms in obesity and T2D-related genes and risk factors of metabolic origin.

## Materials and methods

### Study population

We recruited individuals born and living in the metropolitan area of Merida, Yucatan, a region of southeastern Mexico. These unrelated individuals served as probands used to recruit extended families for the Genetics of Metabolic Disease in Mexico (GEMM) family study (Bastarrachea et al., 2007, 2012). Families were recruited based on family size rather than on any disease status.

### Phenotyping

All phenotypes studied constitute risk factors for T2D and obesity. All participants gave written informed consent to participate in this study. The study was approved by the ethical committee of the School of Medicine, Autonomous University of the Yucatan.

The clinical examination included basic anthropometric measures. Blood samples were collected after an overnight fast of at least 12 h. Stature was measured to the nearest centimeter, and weight was measured to the nearest 0.1 kg, with the subject in light clothing and without shoes. BMI (kg/m^2^) was calculated from this data. Waist circumference was measured to the nearest centimeter with a steel tape measure placed midway between the highest point of the iliac crest and the lowest point of the costal margin. The systolic (first phase) and diastolic (fifth phase) blood pressures were measured to the nearest millimeter Hg with a manually operated sphygmomanometer on the right arm of the seated participant. Three readings were recorded for each individual, and the subject's blood pressure was defined as the average of the second and third readings. Serum samples were obtained from whole blood after clotting.

Upon arrival at Texas Biomedical Research Institute (TBRI), in San Antonio, TX, samples were inventoried. Biochemical phenotypes measured included glucose, insulin, interleukin 6 (IL-6), interleukin 1 beta (IL1β), tumor necrosis factor alpha (TNFα), leptin, and adiponectin. Most of the biochemical phenotypes were analyzed on a Luminex 100 IS platform, consisting of an advanced optometric flux designed to analyze up to 17 different cytokines and chemokines in less than 2 h in ~25 μ L of plasma. An automated clinical chemistry analyzer was used as well as an Immulite 1000, which run ELISA and RIA analysis using ~10 μ L of serum.

### SNP selection

A total of 39 SNPs from 10 genes were selected based on their associations in previously reported GWA studies across various populations (Hirschhorn and Daly, 2005). These genes are adiponectin (*ADIPOQ*) (Takahashi et al., 2000), adiponectin receptors 1 and 2 (*ADIPOR1* and *ADIPOR2*) (Yamauchi et al., 2003; Crimmins and Martin, 2007), proprotein convertase subtilisin/kexin type 1(*PCSK1*) (Choquet et al., 2013), cannabinoid receptor 1 (*CNR1*) (Osei-Hyiaman et al., 2005), fat mass and obesity associated (*FTO*) (Frayling et al., 2007), glucokinase *(GCK*) (Matsutani et al., 1992; Wang et al., 2013), potassium inwardly-rectifying channel, subfamily J, member 11 *(KNCJ11)* (Olson and Terzic, 2010), solute carrier family 30 (zinc transporter), member 8 (*SLC30A8*) (Chimienti et al., 2006) *and* transcription factor like7-like 2 (*TCF7L2*) (Jin and Liu, 2008).

### SNP genotyping

DNA was isolated from frozen buffy coats using organic solvents. Single nucleotide polymorphisms (SNPs) were typed using the multiplex VeraCode technology from Illumina according to the manufacturer's protocol (Illumina, San Diego, CA). Details of the technology are given in Voruganti et al. (2010). Briefly, the technology is based on allele-specific primer extension. Raw data, consisting of intensities of fluorescence, were imported into the analysis software Bead Studio (Illumina). Cluster calls were checked for accuracy and genotypes were exported as text files for further use in association analysis. Replica samples were included as controls for genotyping and allele calling consistencies.

### Statistical analyses

Descriptive statistics and sex-specific differences (Students *t*-test) were calculated using statistical package for social sciences (SPSS, version 10.0; SPSS Inc., Chicago, USA). Genotype frequencies for each SNP were calculated using a maximum likelihood estimation method and were tested for departures from Hardy-Weinberg equilibrium. Estimates of linkage disequilibrium (LD) between SNPs were determined by calculating pair-wise D' and r2 statistics. As a first step in investigating the association between the SNPs in candidate genes and variation in diabetes-related phenotypes, we employed a measured genotype analysis (Boerwinkle et al., 1986), as implemented in the software package sequential oligogenic linkage analysis routines (SOLAR) (Almasy and Blangero, 1998). This approach extends the classical variance components-based biometrical model to account for both the sporadic effects of unrelated individuals and the main effects of SNP genotypes. For each SNP, we compared this saturated model with a null model in which the main effect of the SNP is constrained to zero. The test statistic, twice the difference in loge (likelihood) between the saturated model and the SNP-specific null, is distributed as a chi-square with one degree of freedom. Based on LD between SNPs, the effective number of SNPs was 32, which was used to calculate the empirical *p*-value (Ma et al., 2012). After accounting for SNPs in high LD, the *p*-value of 0.0014 was considered significant.

The T2D and obesity phenotypes were the dependent variables and SNPs were modeled using an additive genetic model (coded 0, 1, and 2 copies of the minor allele). SNP by environment interaction term was added as an additional covariate to the model which was originally adjusted for age, sex, age ^*^ sex age^2^, age^2^ sex (interaction of age with sex, age squared, and interaction of age squared with sex) and the SNP. (Age squared was included to model possible non-linear effects such as accelerated changes in later life, etc. The interactions model possible differential effects of age in men and women, etc.) All environments (internal and external) were modeled as dichotomous variables.

## Results

The study was conducted in 259 (149 women and 110 men) unrelated individuals from the GEMM Family Study. Sex-specific distribution showed significant differences in adiposity-related measures such as body weight, waist circumference, total body fat, leptin, and adiponectin. Systolic blood pressure was also significantly different between men and women, with men having higher pressure. Men also had higher body weight and waist circumference. However, women had significantly higher values of body fat, leptin, and adiponectin. Sex-specific descriptives are shown in Table 1.

**Table 1:** **Descriptive statistics of phenotypes used in the study**.

**Phenotype**	**Mean (SD)**			***p*-value**
	**All**	**Women**	**Men**	
N	259	149	110	
Age (yrs)	30.64 (11.0)	31.57 (11.04)	29.37 (10.88)	0.113
Body weight (kg)	68.82 (10.1)	62.76 (13.1)	77.04 (13.9)	<0.001
BMI (kg/m^2^)	27.18 (5.2)	26.85 (5.6)	27.63 (4.4)	0.226
Waist circumference (cm)	85.93 (13.1)	82.85 (13.7)	90.09 (11.0)	<0.001
Body fat (%)	28.83 (8.7)	32.13 (8.4)	24.35 (7.1)	<0.001
Glucose (mg/dl)	81.88 (9.0)	81.21 (8.3)	82.93 (7.6)	0.162
Leptin (ng/ml)	13.48 (9.6)	18.62 (9.0)	6.61 (5.02)	<0.001
Insulin (μIU/ml)	20.05 (8.5)	19.80 (8.0)	20.40 (9.1)	0.574
IL1B (pg/ml)	1.54 (1.5)	1.55 (1.5)	1.51 (1.4)	0.825
IL6 (pg/ml)	6.47 (12.7)	7.15 (12.1)	5.55 (13.6)	0.322
TNFα (pg/ml)	7.29 (3.0)	7.25 (3.1)	7.34 (2.9)	0.726
Adiponectin (μg/ml)	12.16 (5.2)	13.50 (5.0)	10.40 (5.1)	<0.001
Systolic blood pressure (mmHg)	113.93 (15.0)	108.83 (14.1)	120.85 (13.2)	<0.001
Diastolic blood pressure (mmHg)	75.20 (10.9)	74.44 (11.0)	76.25 (10.6)	0.186

^*^P-values describe the level of significance when comparing men and women for the given phenotypes. P < 0.004 indicates significant difference between men and women (p < 0.05 with Bonferroni correction for 12 comparisons).

Of the total 39 SNPs genotyped, 7 were located within or near *TCF7L2*, 5 from *FTO*, *PCSK1*, and *ADIPOQ*, 4 from *SLC30A8* and *ADIPOR2*, 3 from *CNR1* and *GCK*, 2 from *KCNJ11*, and one from *ADIPOR1*. One SNP, *ADIPOR2*m63422, was dropped from our study due to genotyping error. Minor allele frequency of the remaining 39 SNPs ranged from 2.2 to 48.6% (Table 2). Nominal associations were found for *CNR1, SLC30A8*, *GCK*, and *PCSK1* SNPs with systolic blood pressure, insulin and glucose, and *for CNR1, SLC30A8, KCNJ11*, and *PCSK1* SNPs with adiponectin and leptin (*p* < 0.05). Association of SNPs rs10485170 of *CNR1* and rs5215 of *KCNJ11* with adiponectin and leptin, respectively, reached a *p*-value of 0.002. After adjusting for multiple testing, the association between plasma leptin levels and rs5219 of *KNCJ11* remained significant (Table 3). Minor alleles of rs10485170 and rs5215 were associated with lower levels of adiponectin and leptin, respectively. In contrast, minor allele of rs5219 was associated with higher levels of leptin (Table 4). The SNP by environment interaction was significant for SNP by sex interaction for rs6469675 of *SLC30A8* and systolic blood pressure (*p* = 0.02) and SNP by BMI interaction for rs2268576 of *GCK* and percent body fat (*p* = 0.03).

**Table 2:** **Genotype frequencies**.

**Gene**	**SNP**	**Major allele**	**Minor allele**	**Minor allele frequency**	**Position of the SNP**
*ADIPOQ*	rs3774262	G	A	23.6	Intron
	rs2241766	A	C	23.6	Coding-synonymous
	rs1063539	C	G	22.7	UTR-3′
	rs1501299	C	A	28.5	Intron
	rs266729	G	C	33.5	Upstream- 5′
*ADIPOR1*	rs2275737	C	A	30.3	Intron
*ADIPOR2*	rs2058112	G	A	6.2	Intron
	rs1044471	G	A	34.0	UTR-3′
	rs10773982	G	A	43.2	Intron
	rs1029629	C	A	45.4	Upstream-5′
*CNR1*	rs10485170	A	G	2.9	–
	rs1049353	G	A	9.5	Coding-synonymous
	rs6454674	A	C	23.8	Intron
*FTO*	rs1421085	A	G	17.8	Intron
	rs7193144	A	G	16.4	Intron
	rs8050136	C	A	16.3	Intron
	rs9939609	T	A	16.8	Intron
	rs17817449	A	C	16.4	Intron
*GCK*	rs1799884	G	A	24.2	Upstream-5′
	rs12673242	A	G	15.1	Intron
	rs2268576	G	A	38.1	Intron
*KCNJ11*	rs5215	A	G	37.1	Missense
	rs5219	G	A	36.7	Missense
*PCSK1*	rs6232	A	G	2.2	Missense
	rs6233	A	G	44.3	Coding-Synonymous
	rs6234	G	C	16.0	Missense
	rs6235	G	C	15.9	Missense
	rs271921	G	A	39.5	Intron
*SLC30A8*	rs6469675	A	G	20.4	Intron
	rs2464592	A	G	26.8	Intron
	rs2466293	A	G	48.6	UTR-3′
	rs13266634	G	A	26.1	Missense
*TCF7L2*	rs7903146	G	A	17.1	UTR-3′
	rs7085532	A	G	18.4	Intron
	rs4506565	A	T	18.5	Intron
	rs7901695	A	G	18.1	Intron
	rs6585194	C	G	24.7	Intron
	rs10885406	A	G	23.8	Intron
	rs290483	A	C	42.5	Intron

**Table 3:** **Measured genotype analysis results**.

**Gene**	**SNP**	**Trait**	***P*-value**
*CNR1*	rs10485170	Adiponectin	0.002
		Systolic blood pressure	0.042
*SLC30A8*	rs6469675	Insulin	0.029
	rs2464592	Adiponectin	0.015
		Systolic blood pressure	0.028
*GCK*	rs12673242	Body weight	0.023
	rs2268576	BMI	0.042
		Body fat	0.025
		Glucose	0.028
		BMI	0.016
		Body fat	0.011
		Glucose	0.028
		Adiponectin	0.034
*KCNJ11*	rs5215	BMI	0.017
		Body fat	0.033
		Leptin	0.002
	rs5219	BMI	0.012
		Body fat	0.022
		Waist circumference	0.039
		Leptin^*^	**0.001**
*PCSK1*	rs6233	Waist circumference	0.047
		Leptin	0.018
	rs271921	Glucose	0.022
		Leptin	0.024

* Significant p-value is in bold.

**Table 4:** **Genotype-specific phenotype means for significant or near significant associations**.

**Phenotype**	**Gene**	**SNP**	**Effect size (%)**	**AA (SD)**	**AG (SD)**	**GG (SD)**
Adiponectin (μg/ml)	*CNR1*	rs10485170	3.8	12.36 (5.3)	9.12 (3.1)	–
Leptin (ng/ml)	*KCNJ11*	rs5215	3.6	14.00 (9.3)	13.48 (10.3)	12.19 (8.3)
Leptin (ng/ml)	*KCNJ11*	rs5219	3.9	12.19 (8.5)	13.34 (10.2)	14.10 (9.3)

## Discussion

The main finding of this study is the association of the *KCNJ11* SNP rs5219 with circulating levels of leptin. *KCNJ11* encodes a K_ATP_ channel kir6.2 that plays an important role in insulin secretory function in pancreatic β-cells (Olson and Terzic, 2010). Interestingly, leptin is known to inhibit insulin secretion by activating these channels in pancreatic β-cells (Spanswick et al., 2000). Leptin and insulin also modulate the K_ATP_ channel function in hypothalamic neurons involved in food intake and body weight (Spanswick et al., 1997; Miki et al., 2001). GWAS have identified several gene polymorphisms to be associated with obesity and T2D risk factors. Our SNPs were selected based on those studies. In the current study, we not only replicated the previously identified association, but also found association of these SNPs with phenotypes not reported in prior GWAS. Besides rs5219 of *KCNJ11*, rs5215 also showed strong association with leptin levels, reflecting the role of leptin in K_ATP_ channel modulation of insulin secretion (Kieffer et al., 1997; Olson and Terzic, 2010).

It is worth mentioning that in our data the rs10485170 SNP from gene *CNR1* was not significantly associated with circulating levels of adiponectin (*p* < 0.002). Previous studies on this gene, which codes for an endocannabinoid receptor 1 that is involved in appetite and energy homeostasis (Despres et al., 2006), have nevertheless shown association with obesity-related phenotypes (Pi-Sunyer et al., 2006; Russo et al., 2007). In rodent models, cannabinoid receptor1 (CB1) inhibits the production and release of adiponectin (Matias et al., 2006). In a study in Romania, SNP rs1049353 was associated with lower adiponectin levels (Dinu et al., 2011). In addition, two human studies did not find evidence of association between *CNR1* expression and plasma adiponectin levels (Lofgren et al., 2007; Pagano et al., 2007). Further research is needed in order to elucidate the link between the polymorphisms of the CNR1 gene and adiponectin levels.

Findings from this cohort study suggest that a particular unrelated population born and living in the Yucatan, a region of the southeast in Mexico, sharing the same environment, could help target genes involved in complex traits. The fact that we were able to obtain positive results for associations previously reported in the literature suggests that the study of this Yucatan population can be very useful in research on genetics of common, complex, highly prevalent diseases.

## Author contributions

Victor M. Hernandez-Escalante, Hugo A. Laviada-Molina, and Fernanda Molina-Segui collected the samples and prepared the database; Shelley A. Cole, Karin Haack, and John W. Kent Jr. processed samples in the laboratory; Edna J. Nava-Gonzalez, Marguerite J. Mezzles, V. Saroja Voruganti, and Juan C. Lopez-Alvarenga performed genetic and epidemiological analyses; Raul A. Bastarrachea, Victor M. Hernandez-Escalante, Edna J. Nava-Gonzalez, and Marguerite J. Mezzles wrote the manuscript; Anthony G. Comuzzie and Esther C. Gallegos-Cabriales proofread the manuscript and incorporated changes and corrections for the final draft.

### Conflict of interest statement

The authors declare that the research was conducted in the absence of any commercial or financial relationships that could be construed as a potential conflict of interest.
